# Incidence of shoulder disorders in a cohort of healthcare workers from 2009 to 2020

**DOI:** 10.1007/s00420-023-01976-7

**Published:** 2023-05-10

**Authors:** Thomas Iavernig, Marta Zanette, Andrea Miani, Federico Ronchese, Francesca Larese Filon

**Affiliations:** 1 Postgraduate School in Occupational Medicine, Unit of Occupational Medicine, University of Trieste, Via Della Pietà 2/2, 34129 Trieste, Italy; 2 Unit of Occupational Medicine, University of Trieste, 34129 Trieste, Italy

**Keywords:** Shoulder, Healthcare workers, Incidence, Cohort, Epidemiology, Occupational

## Abstract

**Purpose:**

To estimate the incidence of shoulder disorders in a cohort of Health Care Workers (HCWs).

**Methods:**

4406 workers employed from 2009 to 2020, were included in the study. Occupational risk factors and jobs were assessed according to working history. Incident cases were defined in case of shoulder pain associated with functional limitations during the medical examination. The Cox regression model was used to calculate the Hazard Ratio (HR) for different work activities, adjusted for age, sex, body mass index, and previous musculoskeletal injuries, using clerks as the reference category.

**Results:**

The incidence rates of shoulder musculoskeletal disorder for men and women were 13.1 for 1000 person-years (CI 95% 10.6–16.3) and 20.1 for 1000 person-years (CI 95% 17.8–22.6) respectively. The adjusted HR was significantly increased with age (1.06, CI 95% 1.05–1.07), outpatient health activities (2.82, CI 95% 1.89–4.219), and wards health activity (2.37, CI 95% 1.68–3.33).

**Conclusion:**

HCWs with high biomechanical risk such as nurses and healthcare assistants had a higher incidence of shoulder disorders. Actions are needed for better prevention in health care assistance.

## Introduction

Work-related musculoskeletal disorders (MSDs) are defined by symptoms in the osteoarticular district that can be related to occupational reasons. These included: nonspecific joint discomfort, persistent pain, and anatomical damage to the musculoskeletal structures involved (Kee and Seo 2007; Bernal et al. 2015; Cherry et al. 2000). They represent one of the main causes of workers’ temporary disability (INAIL, 2012) and a major cause of years lived with disability (YLDs) along with mental and behavioral disorders, diabetes, and endocrinological diseases (Vos et al. 2021), a fact that is certainly aggravated by the general aging of Italian working population and the inversion of the demographic pyramid (Palmer and Goodson 2015), (ISTAT, 2020).

The burden of MSDs disorders goes beyond the health context and affects the socioeconomic, personal, and collective sphere of the worker; often, these disorders cause long periods of absence from work, limitation of fitness for the specific task, and, in the most serious cases, can contribute to worker retirement (Bernal et al. 2015). In Europe, it is estimated that the cost of lost productivity due to osteoarticular disorders accounts for about 2% of the GDP (Gross Domestic Product) of member states (Bevan 2015).

The prevalence of these disorders is probably underestimated (Hodgetts et al. 2021). In our Country, interest in this subject has only emerged over the past few years, and more specifically, after the introduction of Legislative Degree 81/2008. Evidence of this comes to us from the number of reports of MSD cases, which appear to be steadily growing since 2005, coming to be today the category of pathologies most reported to the insurance agency (INAIL, Istituto Nazionale Assicurazione contro gli Infortuni sul Lavoro).

Important occupational exposures for shoulder joint pathologies include: manual handling (heavy lifting, pushing, pulling, holding, carrying), working above shoulder height, repetitive work, vibration, and working awkward postures (Linaker and Walker-Bone 2015).

Biomechanics of the shoulder is quite special, it allows a decidedly wide ROM (Range of Movement) when compared with other joints of the human body, and it is supported decisively by muscles and tendons (Lugo et al. 2008), which, in addition to being exposed to a greater risk of acute trauma, can go into premature underwear. The shoulder is a complex system formed in turn by four joints: the sternoclavicular, acromioclavicular, glenohumeral, and scapulothoracic. From a biomechanical point of view, incorrect overloading can lead to injury in each of these joints.

A critical issue in the evaluation of MSDs is the high prevalence of MSDs in the general population (Östör et al. 2005), a fact known in the literature, and which obliges us to consider musculoskeletal disorders in general, and more specifically those affecting the shoulder joint, as a work-related disease. It turns out that numerous personal risk factors can contribute to and influence both the genesis and the course of the disorder, among which we can include: age, body mass index (BMI), physical activity, previous joint trauma, systemic diseases such as diabetes, dysmetabolic and/or rheumatologic diseases (Herin et al. 2014), cigarette smoking (Abate et al. 2013), as well as the role played by psychosocial and organizational work factors (Bernal et al. 2015).

Obviously, in workers who perform manual handling of loads, one of the major causes of disability is not only the process of joint wear and tear brought about by overload and age, but also the consequences of injuries (Fulton-Kehoe et al. 2000). It should also not be forgotten that the muscle structure also plays an important role in bearing the biomechanical stress on the joints of the upper limb (Lefèvre-Colau et al. 2018).

Often, an injury to the musculoskeletal system can impair work performance even in the long term, causing work re-organization, resulting in loss of the professionalism acquired by the worker during his or her working career. In literature, this figure is summarized through the estimation of years of productivity lost (YPLs), a calculation that allows guiding public health choices (Lefèvre-Colau et al. 2018), given the economic, as well as social, weight that these represent for the Country.

Based on these reflections, and from the perception of a high occurrence of this type of disorder in the daily practice of the Medical Officer, we wanted to proceed with the estimation of the impact of MSDs affecting the shoulder joint in healthcare workers referred to the Unit of Occupational Medicine of Trieste in the period 2009–2020.

The clinical question underlying this study was to quantify the incidence of MSDs in the shoulder joint and to ascertain the main characteristics of the population affected. In particular, we wondered whether the biomechanical risk of Health Care Workers (HCWs) could increase the incidence of these disorders in different job titles. We know that the category of HCWs represents a particular group in terms of both type of exposure and composition (Bernal et al. 2015), and the biomechanical stress to which an HCW is subjected differs greatly from the manual handling of loads typical of tasks classically considered in risk assessments (16; 17).

Shoulder musculoskeletal disorders occur in different occupational categories (18; 18) and more specifically in HCWs (Kee and Seo 2007), (Doorn et al. 2021). However, most studies focus on lumbosacral spine disorders, such as herniated discs and spondylodiscitis (Yanik et al. 2022).

As reported in the literature (Heiden et al. 2013), one of the biases of retrospective studies is the difficulties in occupational exposure’s definition, which is often obtained through approximate estimates. Moreover, most studies based the case definition on the subjective perception of complaints often noted through questionnaires, and rarely on the simultaneous medical examination. On the other hand, we know that clinical objectivity often does not correlate with anatomical alterations, and self-administered questionnaires for symptom detection do not have the same objective value as a medical examination. To give greater methodological robustness to the study, therefore, we chose to consider as a case the worker with shoulder symptoms and a positive medical examination for shoulder diseases.

## Materials and methods

Since 2009, the Institute of Occupational Medicine in Trieste has been equipped with computer-writing software for recording risk health in the conduct of Health Surveillance performed according to the Italian Low 81/2008 before the start of the job task and periodically every 2 years or in case of workers’ request for symptoms work-related.

Subjects with signs and symptoms of shoulder diseases at the first medical examination were excluded from the analysis. Incident cases were defined in presence of pain and functional limitation at the shoulder joint, accompanied by the finding of positivity of an objective medical examination using clinical tests for shoulder pathology (Heiden et al. 2013), or/and the presence of a diagnosis of osteoarticular pathology certified by a specialist physician, with the indication of the year of reporting. Additional data collected were age, weight and height, body mass index (BMI), length of employment, history of work-related injuries, department and job title at first and last visit, and, for cases only, year of diagnosis and length of employment at the time of diagnosis. The biomechanical risk was stratified according to task (aggregated into six main categories) and the department to which it belongs.

We calculated BMI for each patient and then divided them into four classes: underweight = BMI < 18.5 kg/m^2^; normal weight = 18.5 kg/m^2^ > / = BMI > 25 kg/m^2^; overweight = 25 kg/m^2^ < / = BMI < 30 kg/m^2^; obesity = BMI > / = 30 kg/m^2^. Jobs were summarized as 1. Administrative or assimilated activity: 2. Outpatient health activities (health care assistant—nurses); 3. Ward health care activity (health care assistant—nurses); 3. Technical health activity (physiotherapist, speech therapist, technicians, etc.); 4. Maintenance or logistics area technical activity; 5. Physicians.

The biomechanical risk classification was performed according to hazard evaluation as 0 = no risk; 1 = low risk; 2 = medium risk; 3 = high risk. The risk assessment, needed for the Italian Law 81/2008, considered the assessment of manual handling of loads carried out according to the specific model proposed by NIOSH (1993), which is able to determine, for each lifting action, the so-called “recommended weight limit” through a calculation that, starting from a maximum liftable weight under ideal conditions, considers the possible existence of unfavorable elements and treats these with appropriate de-multiplication factors. For the patients’ handling was used the method proposed by MAPO (Movement and Assistance for Hospitalized Patients) (Battevi and Menoni 2012), while for flat displacement, towing and loads thrust the Snook & Ciriello method was applied (UNI ISO 11228–2). Risk classification put together all evaluations performed.

Data analysis was performed using STATA 14 software (StataCorp. Texas, USA). Initially, a descriptive sample analysis was performed, Continuous data were summarized as mean and standard deviation and were compared using Students’ *t*-test. Categorical data were summarized as numbers and percentages and were compared using chi-square statistics.

Incident cases were calculated considering new cases with shoulder diseases by person-years at risk (× 1000). The Cox regression model was used to calculate the Hazard Ratio (HR) and 95% confidence intervals (95%CI) for different work activities, adjusted for age, sex, body mass index, and previous musculoskeletal injuries, using clerks as the reference category. Factors significantly associated to shoulder pain in univariate analysis were inserted in the model. Workers with less than 1 year of exposure were excluded from the analysis as well as cases with data missing. The level of significance was set for p < 0.05.

## Results

Cohort characteristics. Table 1 shows the cohort’s characteristics. The majority of the population studied were women (66.4%) Three hundred forty-nine cases (7.9%) had shoulder diseases before the start of working in our hospital while for 426 (10.5%) the disorder arose in the course of work. Forty-seven % of the group was involved in ward activities and 49.6% were classified as involved in job tasks with high biomechanical risk. Six-hundred and thirty workers (15.5%) had had an injury that involved musculoskeletal apparatus during work.

**Table 1:** Cohort characteristics (total n. 4406)

Characteristic	*N*
Age in years, mean (SD) Age classes, years *n* (%) < 40 40–49 50–59 ≥ 60	48.3 (12.6) 1144 (28.2) 950 (23.4) 1030 (25.4) 933 (23.0)
Gender, *n* (%)	
Female	2694 (66.4)
Male	1363 (33.6)
Seniority of work in years, mean (SD) Seniority of work classes, years *n* (%) < 1 1–10 11–20 > 20 Cases, *n* (%)	14.1 (13.20) 796 (19.6) 1197 (29.5) 894 (22.04) 1170 (28.84)
Healthy	3631 (81.6)
Prevalent cases	349 (7.9)
Incident cases BMI, *n* (%)	426 (10.5)
Underweight	124 (3.1)
Normal weight	2287 (56.4)
Overweight	1107 (27.3)
Obesity	539 (13.3)
Job categories, *n* (%)	
Administrative staff	760 (17.3)
Nurse and assistant nurses	
-in outpatients	307 (7.0)
-in ward	2070 (47.0)
Sanitary Technician	439 (10.0)
Logistic Technician	232 (5.3)
Physician	598 (14.0)
Biomechanical Risk Groups, *n* (%)	
No Risk	1392 (31.6)
Low	628 (14.3)
Medium	199 (4.5)
High	2187 (49.6)
Injuries, *n* (%)	
No	2901 (71.5)
Musculoskeletal	630 (15.5)
Other	526 (13.0)

## Analysis of incidence

The incidence rate for shoulder disease was calculated considering a total of 19,898 person-years (Table 2).

**Table 2:** Crude incidence rates per 1000 person-year and 95% confidence intervals) by gender, age classes, seniority of work classes, biomechanical risk job categories, BMI classes, and occupational injury

	Incident cases	Person- years	Rate (per 1000 person-years)	95% CI
Overall	356	19,898	17.9	16.1–19.8
Gender				
Female	274	13,645	20.1	17.8–22.6
Male	82	6253	13.1	10.6–16.3
Age classes (years) < 40 40–49 50–59 ≥ 60 Seniority of work classes, years *n* (%) < 1 1–10 11–20 > 20 Biomechanical risk	29 73 145 109 0 67 141 148	2852 5255 6771 5020 – 4784 7130 7981	10.2 13.9 21.4 21.7 – 14.0 19.8 18.5	7.1–14.6 11.0–17.5 18.2–25.2 18.0–26.2 – 11.0–17.8 16.8–23.3 15.8–21.8
No risk	67	6708	10.0	7.9–12.7
Low	55	3096	17.8	13.6–23.1
Medium	18	839	21.5	13.5–34.1
High	216	9255	23.3	20.4–26.7
Injuries				
No	197	13,028	15.1	13.2–17.4
Musculoskeletal	110	3607	30.5	25.3–36.8
Other	49	3263	15.0	11.3–19.9
Job categories				
Administrative staff	36	3282	11.0	7.9–15.2
Nurse and assistant nurses				
-in outpatients	43	1374	31.3	23.2–42.2
-in wards	216	9265	23.3	20.4–26.6
Sanitary technician	29	1989	14.6	10.13–21.0
Logistic technician	11	1007	11.0	6.0–19.7
Physician	21	2981	7.0	4.6–10.8
BMI				
Underweight	8	581	13.8	6.9–27.5
Normal weight	194	10,933	17.7	15.4–20.4
Overweight	96	5552	17.3	14.2–12.1
Obesity	58	2832	20.5	15.8–26.5

For the entire cohort, the incidence rate was 17.9 cases per 1,000 person-years (CI 16.1 – 19.8), higher for women (20.1 cases per 1,000 person-years 95%CI 17.8 – 22.6,) compared to men (13.1 cases per 1,000 person-years 95%CI 10.6 – 16.3). The incidence of shoulder diseases increased in relation to age classes until 50 years and class of biomechanical risk in a linear function from 10 cases per 1,000 person-years in workers with no risk to 23.3 cases per 1,000 person-years for subjects exposed to high biomechanical risk. A higher incidence was found for health care activities, higher for workers occupied in outpatients (31.3 cases per 1,000 person-years), and lower for clerks (11 cases per 1,000 person-years). The increase in BMI was associated with an increase in incidence only for obese workers.

The results of the multivariate Cox regression are summarized in Table 3.

**Table 3:** Multivariable Cox regression analysis of factors associated with shoulder diseases. Results are reported as Hazard Ratios and 95% Confidence intervals)

	HR	95% CI	*P*-value (*p* < 0.05)
Age	1.06	1.05–1.07	0.000
Gender Female Male	1 0.76	1 0.59–1.00	0.046
Job			
Administrative staff	1		
Nurse and assistant nurses			
-in outpatients	2.82	1.89–4.21	0.000
-in wards	2.37	1.68–3.33	0.000
Sanitary Technician	2.40	0.87–2.25	0.163
Logistic Technician	1.24	0.69–2.26	0.465
Physician	0.74	0.43–1.26	0.263
Injuries No	1	0.88–1.15	0.970
Musculoskeletal	1.48	1.17–1.88	0.001
Other BMI Under weight	0.83 1	0.60–1.14	0.249
Normal weight	1.15	0.57–2.35	0.680
Overweight	1.10	0.53–2.27	0.800
Obesity	1.12	0.53–2.36	0.762

Shoulder diseases increased significantly with age (HR 1.06, 95% CI 1.05–1.07) and male had less problems compared to females (HR 0.76, 95% CI 0.59–1.00). Nurses and assistant nurses involved in outpatients and wards had an increased risk (HR 2.82, 95% CI 1.89 – 4.21 and HR 2.37, 95% CI 1.68 – 3.33, respectively). Workers with shoulder diseases presented an increased risk to have had an occupational injury that involved the musculoskeletal districts.

## Discussion

Our study investigated the incidence of shoulder diseases in the cohort of HCW in Trieste Hospital from 2009 to 2020 and found a significant increase in incidence in relation to age, a higher risk for women and for nurses and assistant nurses involved in both wards and outpatients department compared to clerks, a higher risk associated to musculoskeletal injuries.

Shoulder diseases increased with age, with a higher incidence in workers with more than 49 years, as reported also in other studies (Luime et al. 2004; Alexopoulos et al. 2003; Engels et al. 1996; Lagerstrom et al. 1995; Larusso et al. 2007; Leighton and Reilly 1995; Trinkoff et al. 2003). However, probably due to a “health working effect” there are also studies that denied an association with age (29; 30), and in longitudinal cohort studies (31; 32) age did not reach statistical significance.

HCWs are at increased risk for developing musculoskeletal disorders for excessive loading during patients' handling and awkward postures (33; 34). The prevalence of chronic pain in shoulders was reported to be 28% in a Danish study on HCW (Andersen et al. 2011) with figures higher than back pain (23%). In our study, the overall prevalence was 18.4% (considering subjects with previous shoulder diseases and incident cases during our study), a value lower than that reported by (Andersen et al. 2011). The difference could be related to case definition and to the fact that in our study data were derived from periodical medical examination and not from questionnaires, for which an overestimation of cases is expected. Moreover, a meta-analysis performed by Govaerds et al. in 2021 (Govaerts et al. 2021) reported a prevalence of 54% considering available literature on musculoskeletal disorders in Europe in the secondary industry, suggesting a higher prevalence of the diseases in the industry compared to the healthcare sector. Studies that considered medical records and a standardized physical examination reported more reduced prevalence (Heiden et al. 2013) compared to the majority of data in the literature that are obtained mainly by self-administered questionnaires.

The overall incidence rate of shoulder disorders was found to be 17.9 cases per thousand person-years. This figure is higher than that reported by general practitioners, estimated in the study by Morrey et al. (Morrey 2012), to be 9.5 cases per 1000 person-years (95% CI 7.9 -11.2 per 1000), a context that is, moreover, very different from ours in terms of population composition. This result is in line with the incidence reported by clerks in our cohort, with 11 cases per 1000 person-years. In another paper published in 2012, the incidence of shoulder disorders that came to the attention of general practitioners in the Dutch health care system was estimated to be 29.3 cases per 1000 person-years, with an increased incidence in women and the 45–64 age group (Greving et al. 2012). Figures higher than that found in our study.

The increased risk for women was confirmed also in our study, in which a protective effect for men was found (HR 0.76; 95%CI 0.59–1.00). Moreover, in our cohort, nurses and assistant nurses are mainly women and their job tasks are characterized by a more strenuous physical exertion with patient handling—including turning, moving, lifting, and repositioning patients. On the opposite, men are involved in lighter job tasks (i.e., physicians). Note that the incidence was similar for nurses and assistant nurses involved in wards and outpatients because in case of musculoskeletal symptoms HCWs were assigned to less demanding work tasks, such as those performed in outpatients.

The increase of incidence by biomechanical risk is clearly shown in our results with 10 cases per 1000 person-years (CI 95% 7.9—12.7) for the “no risk” category to 23.3 cases × 1000 person-years (95%CI 20.4 – 26.7) in the high-risk category.

Despite the wide literature available on musculoskeletal disorders in HCWs (Greving et al. 2012), no data on the incidence of shoulder diseases on a long-time follow-up has been published, to the best of our knowledge. Our study confirmed the role of age, sex, of job tasks according to risk assessment. Moreover, our results highlight the need to improve the preventive approach for nurses and nurse assistants reducing more biomechanical demanding job tasks and increasing the use of methods that reduce manual handling of patients. On this aspect, as reported by Wåhlin et al. (2021) the use of aids such as lift and slip sheets, is not constant and more efforts are needed to perform adequate training to convince operators of the usefulness of using them (Wåhlin et al. 2021). At the same time, perhaps, the active involvement of workers in the risk assessment process could increase their knowledge and compliance with the need to follow good practices on patient handling.

## Strengths and limitations

The strengths of our work are a) the cohort design and the long follow-up; b) the criteria for case definition, using symptoms and medical objective examination; c) the third is the use of a standardized procedure for exposure definition derived from the hazard evaluation performed in workplaces in the period considered.

Moreover, our study has some weaknesses, the most important is the retrospective design and the absence of information about medication intake, sports activity, and smoking habits, all of which could be independent risk factors for the occurrence of shoulder disorders.

## Conclusions

Our study demonstrated in a cohort of HCWs followed from 2009 to 2020, a significantly higher incidence of shoulder diseases in older women working as nurses and assistant nurses. More efforts are needed to improve the way of work, reduce loading, improve the use of lift or slip sheets, and increase workers’ compliance with safety procedures.

Age-specific priorities for preventive actions should be reconsidered, including optimization and changing of detrimental working conditions. That will help to reduce the years lived with a disability because of shoulder disorders (Vos et al. 2021).

## Data Availability

The datasets generated during and/or analysed during the current study are available from the corresponding author on reasonable request.
